# Characterisation of a panel of anti-tetanus toxin single-chain Fvs reveals cooperative binding

**DOI:** 10.1016/j.molimm.2010.02.020

**Published:** 2010-06

**Authors:** Nathan Scott, Omar Qazi, Michael J. Wright, Neil F. Fairweather, Mahendra P. Deonarain

**Affiliations:** Division of Cell and Molecular Biology, Faculty of Natural Sciences, Imperial College London, Exhibition Road, London, SW7 2AZ, UK

**Keywords:** B-TeNT-Hc, biotinylated TeNT-Hc, bp, base pairs, BSA, bovine serum albumin, CDR, complementarity determining region, CNS, central nervous system, CRAb, chelating recombinant antibody, ELISA, enzyme linked immunosorbant assay, *E. coli*, *Escherichia coli*, GT1b, trisialoganglioside, HRPO, horse radish peroxidise, IMAC, immobilised metal affinity chromatography, IPTG, isopropyl-β-d-thio-galactopyroanoside, *K*_A_, association equilibrium constant, *K*_D_, dissociation equilibrium constant, *k*_on_, on-rate, *k*_off_, off-rate, kbp, kilobase pairs, kDa, kilodaltons, mAb, monoclonal antibody, MW, molecular weight, mPBS, milk protein in PBS, OD, optical density, PAGE, polyacrylamide gel electrophoresis, PBS, phosphate buffered saline, PCR, polymerase chain reaction, scFv, single-chain Fv, SPR, surface plasmon resonance, TeNT, tetanus neurotoxin, TeNT-H_c_, C-terminal domain of tetanus toxin heavy chain, TeNT-H*c*C, C-terminal sub-domain of TeNT-H_c_, TeNT-H*c*N, N-terminal sub-domain of TeNT-H*c*, VH, variable domain heavy chain, VL, variable domain light chain, Tetanus toxin, Phage display, Affinity, Cooperativity, Neutralisation

## Abstract

An approach for enhancing antibody affinity is to engineer Chelating Recombinant Antibodies (CRAbs) which consist of two tandemly linked single-chain Fvs (scFvs) that bind to distinct non-overlapping epitopes on the antigen molecule leading to a synergistic decrease in *K*_D_. In order to develop this technology, the aim of this present study was to identify scFvs which can simultaneously bind to the tetanus toxin heavy chain C-terminal sub-domain (H_c_), characterise their bio-physical properties and determine their functional efficacy.

Over 50 antibodies specific for H*c* were isolated from a human scFv phagemid library and found to bind specifically to the C-terminal sub-domain of H_c_ (H_c_C clones), the N-terminal sub-domain (H*c*N clones) or junctional epitopes on the whole H*c* fragment only (H*c*J clones). Fifteen clones were assayed in a pairwise competition binding study. The revealed, with few exceptions, that H_c_C clones were able to simultaneously bind to the toxin with H_c_N or H_c_J clones. All other combinations competed for binding. Interestingly, we also observed cooperative binding with many non-competing scFv pairings which may impact upon the binding mechanism of CRAbs. We found that 14/15 clones neutralised toxin activity in a ganglioside binding assay and this effect was strongly related to affinity. This included clones that did not bind to the H_c_C sub-domain which is responsible for direct interaction with gangliosides on nerve cells. For 7 scFvs that underwent further characterisation we found broad variations in propensity for multimerisation, affinity and potency.

The diverse array of clones characterised in this paper can be used to construct CRAbs and will prove useful in further characterisation of toxin biology and in measuring the effects of polyclonal antibody therapy.

## 1 Introduction

In developing countries, tetanus remains a major cause of mortality with between 800,000 and 1,000,000 deaths annually (Dietz et al., 1996). The symptoms of disease are caused by tetanus toxin, a protein released by the bacterium *Clostridium tetani* usually upon infection of a wound (Farrar et al., 2000). The toxin migrates from the periphery to the central nervous system (CNS) by retrograde axonal transport and trans-synaptic spread (Lalli et al., 2003a). Within inhibitory neurones of the CNS, the toxin undergoes vesicular endocytosis and drastically reduces the secretion of the inhibitory neurotransmitter γ-aminobutyric acid into the synaptic cleft (Collingridge and Davies, 1982), resulting in a spastic paralysis.

**Fig. 1:**
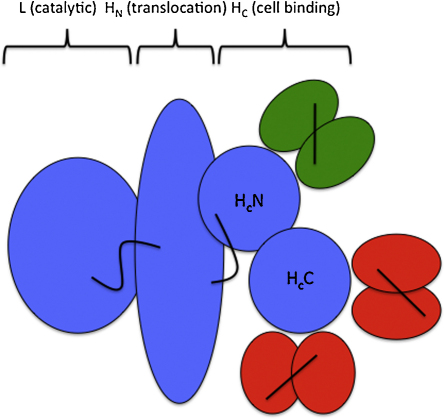
Tetanus toxin. Tetanus neurotoxin is a 150 kDa tripartite toxin with a 50 kDa light chain which exhibits zinc endopeptidase activity and a 100 kDa heavy chain (H). The Hc domain can be further derivatised into two topologically distinct sub-domains termed H*c*C and H*c*N denoting C and N-terminal portions respectively. The panel of scFvs used in this study specifically binds the N-terminal sub-domain (clones N1 through N5), C-terminal sub-domain (C1 through C5) or the whole Hc fragment only (J1 through J5).

Tetanus neurotoxin (TeNT) is a 150 kDa protein which is post-translationally cleaved to produce a 50 kDa light chain (L) joined by a disulphide bond to a 100 kDa heavy (H) chain (Fig. 1). The H chain contains two functional domains, each of approximately 50 kDa. The N-terminal domain (H_N_) is required for the pH-dependent penetration and translocation of the catalytic L chain across the vacuolar membrane into the neuronal cytosol, an activity probably involving formation of ion channels in lipid bi-layers (Beise et al., 1994; Blaustein et al., 1987; Gambale and Montal, 1988; Hoch et al., 1985). The C-terminal domain of the H chain (H_c_) mediates neuronal cell binding, receptor mediated endocytosis and retrograde trafficking activities of the holo-toxin (Halpern and Neale, 1995). The H_C_ domain is completely non-toxic (Fairweather et al., 1986) and can partially neutralise the toxicity of the tetanus neurotoxin *in vivo* by competing for neuronal binding sites (Bizzini et al., 1977; Lalli et al., 1999). Binding of H_c_ to neuronal cells occurs *via* a low affinity interaction with highly abundant gangliosides and a second highly specific subsequent interaction with an as yet unidentified proteinaceous receptor (Montecucco et al., 2004).

The crystal structure of H_c_ shows it contains distinct N-terminal (H_c_N) and C-terminal (H_c_C) sub-domains (Emsley et al., 2000; Umland et al., 1998). H_*C*_N is composed of two seven-stranded β-sheets coupled in a jelly-roll motif, which may play a role in the intracellular sorting of the tetanus toxin (Lalli et al., 2003b). H_C_C exhibits a modified β-trefoil structure with a six-stranded β-barrel, a motif found in recognition and binding proteins (Umland et al., 1998). TeNT contains ganglioside binding activity which structural studies revealed is mediated through the H_c_C domain (Fotinou et al., 2001).

Several studies have characterised antibodies to TeNT. A panel of 57 mAb molecules raised against tetanus toxoid or toxin fragments unveiled 20 different epitopes on the holo-toxin toxin (Volk et al., 1984). Another study (Fitzsimmons et al., 2000) characterised 13 monoclonal IgGs recognising 5 different epitopes of H_C_, 2 of which were overlapping. In this study, with the exception of one antibody, all were shown to reduce ganglioside binding of the toxin to different extents. Interestingly the other antibody actually caused an increase ganglioside binding. In a study of the diversity of the human immune response against the tetanus toxoid, over 100 different toxin specific antibody clones were isolated from a single individual (Poulsen et al., 2007).

Recombinant antibody fragments can be useful diagnostic and immunological tools as well as therapeutic agents. The single-chain variable fragment (scFv) format (Bird et al., 1988; Huston et al., 1988) consists of an immunoglobulin variable heavy (VH) and variable light (VL) domain joined with a flexible hydrophilic linker such as (G_4_S)_3_. ScFvs are amenable to bacterial expression and can be selected from large naïve combinatorial antibody libraries using techniques such as phage display and engineered into a variety of binding structures (Hoogenboom, 2005). One example is the chelating recombinant antibody (CRAb)-a type of tandem scFv (Neri et al., 1995). These are bi-specific antibodies with scFv components cognate for two adjacent yet non-overlapping epitopes on the same monomeric antigen molecule. The two scFvs are joined with a linker of sufficient length to allow dual epitope binding which results in a synergistic improvement in binding affinity compared to the components alone. The CRAb format can be derived through rationale design (Neri et al., 1995) or selection from combinatorial libraries of tandem-scFv molecules (Wright and Deonarain, 2007). A pre-requisite to both approaches requires the identification of scFv pairs capable of simultaneous binding to a target molecule as separate entities.

The aim of this study was to isolate and characterise antibodies that bind to the H_c_ domain of TeNT in order to (1) identify antibody pairs able to simultaneously bind to the toxin with a view to making CRAbs of possible therapeutic utility, (2) elucidate further the relationship between affinity and toxin neutralisation and (3) to further characterise the mechanism of action of TeNT. Here we have isolated over 50 different scFvs against H_C_. Of the 15 chosen for detailed analyses, 14 have ganglioside binding neutralising properties which are affinity related and some scFvs exhibit positive cooperativity in binding.

## 2 Materials and methods

### 2.1 *Bst*NI antibody fingerprinting

The scFv DNA sequence inserts were amplified from pCANTAB6 by PCR using primers LMB3 (5′-CAGGAAACAGCTATGAC-3′) and FDSEQ1 (5′-GAATTTTCTGTATGAGG-3′). Reactions included primers at a concentration of 10 pmol/μl, an equimolar mixture of dNTPs (200 μM final concentration) and 25 mM MgCl_2_. TAQ polymerase (Sigma) (5 u) was added along with the appropriate buffer supplied by the manufacturer and nuclease free water. The PCR reaction mixture was aliquoted into 25 μl volumes and 1 μl of *Escherichia* *coli* XL1-Blue scFv phagemid clone overnight culture was added. The reaction mixture was then subjected to repeated rounds of heating and cooling in a thermocycler. The cycling conditions were 25 cycles of 1 min at 96 °C, 45 s at 50 °C and 45 s at 72 °C. A final extension of 10 min at 72 °C was also included.

Restriction fragment length polymorphism (RFLP) analysis of the scFv inserts was carried out by adding 5 u of *Bst* NI to the 25 μl PCR reaction mixture along with NEB buffer 2 (50 mM Tris–HCl, 100 mM NaCl, 10 mM MgCl_2_, 1 mM dithiothreitol, pH 7.9 (25 °C)) and BSA as per manufacturer's instructions. The digests were incubated at 60 °C overnight. The digested DNA was loaded onto a 3% agarose gel and separated at 100 V. The resulting RFLP patterns were visualised and compared by eye.

### 2.2 Expression of TeNT recombinant proteins in *E.coli*

The TeNT-H*c*, TeNT-H_c_C and TeNT-H_c_N expression cassettes were encoded on a pET28a (Promega) (pKS1) expression vector under the control of a T7 promoter. Their expression and purification is as previously described (Sinha et al., 2000). *E. coli* BL21 (DE3) (pKS1) cells transformed with TeNT-H_c_ in pET28a were grown in 0.5 L cultures of 2TY containing 0.1% glucose and 50 μg/ml kanamycin at 37 °C with shaking at 250 rpm. Expression was induced with 1 mM isopropyl-1-thio-β-d-galactopyranoside (IPTG) at an OD_600_ of approximately 0.8 for a further 4 h. Cells were lysed by French press into 100 mM sodium phosphate buffer containing 300 mM NaCl, 10 mM imidazole and a protease inhibitor cocktail tablet (Roche) (pH 7.8). The lysate was sonicated for 5 min with 15-s pulses followed by 15-s gaps in order to break up genomic DNA causing sample viscosity. Typically, 500 ml of original culture volume would result in 25 ml of cell lysate to be applied to an IMAC (immobilised metal affinity chromatography) column.

### 2.3 Expression of scFv proteins in *E. coli*

*E. coli* HB2151 transformed with scFv genes in phagemid vector pCANTAB6 were grown in 1 L cultures of 2TY media containing 100 μg/ml carbenicillin and 0.1% glucose at 30 °C with shaking at 250 rpm. Expression was induced with 1 mM IPTG at an OD_600_ of approximately 0.8 for overnight at 30 °C with shaking at 300 rpm. Culture supernatant was clarified by centrifugation at 9000 rpm for 1 h at 4 °C, concentrated to one-tenth its original volume using a Vivaflow 200 10 kDa MWCO dia-filtration cassette (Vivascience) and then exhaustively dialysed into PBS pH 7.4. Bacterial cells were also used to obtain periplasmic scFv material. The cell pellets were resuspended in ice-cold periplasmic extraction buffer containing 500 mM sucrose, 100 mM Tris and 1 mM EDTA, pH 8.0. The volume of buffer used was one-tenth the final volume. The bacteria were vortexed for 10 s every 5 min for 20 min in order to break open the outer membrane. Spheroplasts were then isolated by centrifugation at 13,000 rpm for 30 min at 4 °C and the supernatant (periplasmic fraction) retained and exhaustively dialysed into PBS pH 7.4.

### 2.4 Purification of TeNT-*H*_c_C, TeNT-*H*_c_N, TeNT-*H*_c_ and scFv proteins by immobilised metal affinity chromatography

Both the recombinant TeNT proteins and the panel of scFv fragments investigated in this study were His-tagged enabling IMAC purification. Typically 1–2 ml of TALON resin (BD Clontech) was incubated with the cell lysate or concentrated supernatant overnight at 4 °C with gentle rolling. After further incubation for 1 h at room temperature, the samples were applied to a 10 ml gravity flow column. For TeNT fragment purification subsequent washing steps were conducted with 100 mM sodium phosphate buffer containing 300 mM NaCl and 25 mM imidazole (pH 7.8). Typically 4 column volumes of wash buffer were used. The TeNT fragments were eluted in 1 ml fractions of 100 mM sodium phosphate buffer containing 300 mM NaCl and 200 mM imidazole (pH 7.8). Fractions were dialysed exhaustively into 100 mM sodium phosphate buffer containing 300 mM NaCl (pH 7.8) at 4 °C, aliquoted, and stored at −80 °C in 5% glycerol. For purification of scFv proteins, the wash step consisted of 4 column volumes of washes with PBS pH 7.4. The scFv proteins were eluted in 1 ml fractions using 200 mM imidazole in PBS (pH 7.4). The scFv proteins were stored in elution buffer supplemented with 5% glycerol. All purified proteins were quantified by Bradford reagent and analysed by reducing 12% sodium dodecyl sulphate-polyacrylamide gel electrophoresis (SDS-PAGE) followed by staining with Coomassie R-250. Western blot was used to confirm the presence of tagged proteins at the correct molecular weight.

### 2.5 Size exclusion chromatography

Size exclusion chromatography (SEC) was routinely used as a subsequent purification step after purification of TeNT-Hc or scFv proteins by IMAC. The TeNT-H_c_ protein (MW = 53 kDa) was excluded using a Superdex-200 column and scFvs (MW = 30 kDa) using a Superdex-75 column both eluted with PBS pH 7.4 at 1 ml/min. Fractions were analysed by SDS-PAGE. Interpolation of elution volume with a standard calibration enabled estimation of apparent molecular weight of fractionated proteins. The ratios of different protein peaks were estimated from measuring the area under the curves of each peak, which takes into account volume as well as concentration.

### 2.6 Chemical biotinylation of TeNT-*H*_c_

Sulfo-NHS-LC-LC-biotin reagent (sulfosuccinimidyl-6-(biotinamido)-6-hexanamido hexanoate-Pierce EZ-Link^®^) was used to minimally biotinylate the purified monomeric TeNT-H*c* fragment at a 1:1 molar ratio of 1 h at room temperature. The free biotinylation reagent was removed using a PD10 desalting column and eluted into 100 mM sodium phosphate buffer containing 300 mM NaCl, 0.05% surfactant p20 and 0.005% sodium azide. This generated predominantly singly biotinylated TeNT-Hc with some free TeNT-H*c*.

### 2.7 Anti-TeNT-*H*_c_ ELISA

A Nunc™ Maxisorb ELISA plate was coated with 100 μl of TeNT-H_c_ in 50 mM sodium phosphate buffer (pH 7.8), at a final concentration of 10 μg/ml. Coating took place for 2 h at 37 °C. After two washes with PBS (pH 7.4), the plate wells were blocked with 200 μl of 3% Marvel™ in PBS (pH7.4) for 2 h at 37 °C and washed three times in PBS (pH 7.4). Unless stated otherwise, all subsequent wash steps consisted of three washes with PBS (pH 7.4) containing 0.1% Tween-20 and three washes with PBS (pH 7.4). For avidin capture, plates were coated with 100 μl of 20 μg/ml avidin in PBS (pH 7.4), overnight at 4 °C and plates were then blocked with 200 μl of 3% Marvel™ in PBS (pH 7.4) for 2 h at 37 °C and washed three times with PBS (pH 7.4). Biotinylated TeNT-H_c_ (10 μg/ml) was added, in a 100 μl volume, to the wells and incubated for 1 h at room temperature followed by standard washes. Subsequently, three types of assay were conducted as follows:

#### 2.7.1 ScFv ELISA

Appropriate wells were incubated with 100 μl of scFv diluted to an appropriate concentration with 3% Marvel™ in PBS (pH 7.4), for 1 h at room temperature. The plate was washed and bound scFv was detected using 100 μl of the murine 9E10 monoclonal antibody (5 μg/ml, CR-UK) in 3% Marvel™ in PBS (pH 7.4). Incubation for the primary detection step was conducted for 1 h at room temperature. After subsequent washing of the plate the wells were incubated with a secondary detection antibody, goat anti-mouse HRPO conjugate (Sigma) at a dilution of 1:10,000 in PBS (pH 7.4), containing 3% Marvel™.

#### 2.7.2 Phage ELISA

Appropriate wells were incubated with 100 μl of phage-scFv for 1 h at room temperature. The phage fusions were diluted with 3% Marvel ™ in PBS (pH 7.4). A dilution series was often used from 1 in 2 in 2-fold steps down to 1 in 1024. After washing of the wells, bound phage was detected using a monoclonal anti-M13 antibody directly conjugated to HRPO (GE Healthcare). The conjugate was used at a dilution of 1:5000 in 3% Marvel™/PBS pH 7.4. A volume of 100 μl of conjugate was incubated for 1 h at room temperature in each well.

#### 2.7.3 Competition ELISA

Coated toxin was pre-incubated with 50 μl of scFv at a concentration of approximately 200 μg/ml in 3% Marvel™ PBS (pH 7.4) for 1 h at room temperature. Fifty microlitre of phage-scFv at two times the desired final limiting dilution in 3% Marvel™ PBS (pH 7.4) was added to the wells already containing pre-incubated scFv, mixed thoroughly and left for 1 h at room temperature. A negative scFv control was included. The plate was washed and phage-detected as above.

For all forms of ELISA described, following addition of the final layer HRPO conjugate detection antibody, a washing step was conducted followed by the addition of peroxidise substrate (POD, Roche). The peroxidise reaction was allowed to proceed as desired and stopped with 1 M HCl. The plate wells were read on an absorbance plate reader at 450 nm.

### 2.8 Surface plasmon resonance binding analysis

Streptavidin (SA) chips were used for all experiments carried out on the BIAcore 3000. The running buffer used was 100 mM sodium phosphate pH 7.4 containing 300 mM NaCl, 0.005% p20 surfactant and 0.005% sodium azide. Biotinylated TeNT-H_c_ (1:1 biotin:toxin ratio) was applied until 700-800 RU was immobilised. Regeneration conditions were a 30 s pulse of 10 mM glycine–HCl pH 2.5 at 30 μl/min. The surface was capable of withstanding around 100 regeneration cycles using these conditions. However, 2 mM HCl containing 150 mM NaCl was needed for higher affinity clones (C1 or C4): the surface was amenable to far fewer cycles with this regeneration buffer. Only monomeric scFv (500–4 nM) was used in kinetic binding experiments, run in duplicate, kinetic scFv binding data were evaluated using the BIAevaluation software (version 4.1; 2003) subtracting reference data using double referencing.

### 2.9 ScFv-phage library selection

The Vaughan library was obtained from Cambridge Antibody Technology (now Medimmune AstraZeneca) (Vaughan et al., 1996) which contains 1.4 × 10^10^ unique scFv clones in the pCANTAB6 phagemid vector.

For production of monoclonal or polyclonal phage fusions, 50 ml of *E. coli* XL1-Blue transformants were grown in 2TY containing 15 μg/ml tetracycline, 100 μg/ml carbenicillin and 1% glucose shaking at 37 °C. At a culture density of OD_600 nm_ = 0.5, Helper phage (VCSM13, Stratagene) was added to the bacteria at an M.O.I. of 20:1 and left at 37 °C with no shaking for 30 min followed by gentle shaking at 200 rpm for further 30 min at 37 °C. The culture was then centrifuged and cell pellets were resuspended in 50 ml of 2TY containing 50 μg/ml kanamycin, 100 μg/ml carbenicillin and then cultured overnight at 30 °C with shaking at 300 rpm. The culture supernatant was isolated by centrifugation and 1/5 volume of ice-cold 20% PEG-8000 containing 2.5 M NaCl was added and incubated on ice for at least 2 h to precipitate the phage particles which was collected by centrifugation. The phage was stored at −80 °C in PBS containing 10% glycerol. Total phage titre was determined using spectroscopy. An OD_270_ of 1.0 approximates to a phage titre of 1.1 × 10^13^ phage particles per 1 ml of neat phage solution for a phagemid of 5 kb (Russel et al., 2004). To determine the infective titre, an infection assay was conducted on *E. coli* strain XL1-Blue with phagemid particles serially diluted 10-fold down to 1 in 1 × 10^8^.

### 2.10 Phage display biopanning

TeNT-H_c_ protein (0.5 ml of a 10 μg/ml solution in PBS) was coated overnight at 4 °C onto a Nunc maxisorp immunotube, rinsed and blocked with 3% milk powder/PBS for 1 h at room temperature. 50 μl of the CAT library was added to 450 μl of 3% milk powder in PBS in a 1.5 ml microfuge tube and was incubated at room temperature for 30 min in order to pre-block the phage. The pre-blocked phage was added to the immunotube and incubated for 90 min at room temperature. The tube was then washed 15 times with PBS/0.05% Tween 20 (PBS/T) followed by 15 rinses with PBS. Phagemids bound to the antigen were then eluted with 0.5 ml of 100 mM glycine pH 2.2 for 15 min at room temperature. A second elution step was then carried out by adding 0.5 ml of 100 mM triethylamine (TEA, Sigma) to the immunotube for a further 15 min at room temperature. The third elution step was the addition of 0.5 ml of exponentially growing *E. coli* TG1 cells (OD_600_ = 0.5–0.8) for 30 min stationary and plated onto a large (150 mm) diameter 2TY agar plate containing 100 μg/ml carbenicillin and 2% (w/v) glucose (2TYCG). The glycine and TEA eluted phagemids were added to 5 ml of exponentially growing TG1 cells for 30 min and similarly plated. To store the eluted phagemid colonies, cells were resuspended in 5 ml of 2TY/10% glycerol and stored at −80 °C. From this, 100 μl of the pooled culture was used to inoculate 25 ml of 2TY containing 100 μg of carbenicillin, 2% (w/v) glucose (2TYCG) to produce phage for the next round using helper phage rescue.

### 2.11 Screening for *H*_c_-binding phagemid clones

*E. coli* TG1 colonies (540 clones) isolated from round 2 of phage display were picked, using sterile toothpicks, into sterile microtitre plate wells containing 120 μl of 2TYAG. Colonies were picked at random and two 96-well microtitre plates were used for each of the phage elution methods; TEA, glycine and bacterial. Eight blank wells were included on each plate in random positions to control for cross-contamination of wells. Microtitre plates were sealed with sterile adhesive barriers and incubated at 37 °C with 200 rpm shaking overnight. The plates were centrifuged at 863 × *g* for 10 min and the supernatant discarded. The bacterial pellets were resuspended in 50 μl of 2TY/50% glycerol (v/v) to make a stock which was then stored at −70 °C and served as a master plate. Before freezing, 10 μl of culture from each well in the master plate was used to inoculate fresh microtitre plates containing 120 μl of 2TYAG in each well. The plates were sealed and incubated at 37 °C with 200 rpm shaking until the appearance of turbidity in the microtitre plate wells. VCSM13 helper phage was then added to each well at an M.O.I. of 10 (assuming an OD_600 nm_ of 1 = 1 × 10^8^ cells/ml) and plates were incubated for 30 min static and 30 min shaking at 200 rpm, both steps at 37 °C. The plates were centrifuged at 863 × *g* for 10 min and the pellets resuspended in 100 μl of 2TY containing 100 μg/ml ampicillin and 50 μg/ml kanamycin. The plates were grown overnight at 30 °C with 250 rpm shaking to produce monoclonal phagemids. The plates were centrifuged at 863 × *g* for 10 min and the supernatants, containing the phagemids, added to fresh microtitre plates containing 50 μl of 9% milk powder/3% PBS. Pre-blocked rescued phagemid solution was then assayed for binding to TeNT-Hc, TeNT-HcC and TeNT-Hc using the ELISA methodology outlined above. The absorbencies of ELISA plates were read at 450 nm in an ELISA plate reader and an OD_450 nm_ reading at 0.2 or above was considered a positive binding result.

### 2.12 Tetanus toxin Hc fragment ganglioside binding assay

A Nunc™ Maxisorp 96-well plate was coated with the ganglioside GT1b (Sigma) at a concentration of 10 μg/ml dissolved in 100% methanol which evaporated overnight. The wells were blocked with 3% Marvel™ milk in PBS (pH 7.4) for 2 h at 37 °C. Next, TeNT-H_c_ fragment was added to each well, at a concentration of 10 μg/ml, diluted in 3% Marvel™ in PBS (pH 7.4). To compare the ability of anti-TeNT-H_c_ toxin scFvs in blocking the binding of TeNT-H_c_ to ganglioside, scFvs were pre-incubated with the toxin for 2 h at 37 °C, prior to addition to the ganglioside. Toxin with or without scFv was incubated with the ganglioside. Ganglioside-bound toxin was then detected with a rabbit polyclonal anti-tetanus toxin IgG (1 in 10,000) and secondary goat anti-rabbit HRPO-IgG conjugate. POD substrate was used to develop the ELISA. Three PBS wash steps were included in between each stage.

### 2.13 Statistical analyses

Statistical assessment including the derivation of means and standard errors was carried out using both Microsoft Excel 2007 with Data Analysis Add tool or the Sigma Plot Software Version 8.0 (Systat Software Inc., London). Where appropriate to achieve statistical significance, experiments were independently repeated at least twice. Within each experiment, test samples and concentrations were assayed in triplicate where appropriate.

Student's *t*-test (Microsoft Excel 2007) was used when comparing two means with standard error. However, for comparison of more than two samples an ANOVA test and Dunnett post-test multiple comparison to positive control (GraphPad InStat 3.0). The data points in ELISA *K*_D_ determinations were fitted, using Sigmaplot v8.0, to a standard saturation binding model conforming to 4-paramter logistic curve with maximum, minimum, hill-slope and EC50 (*K*_D_) which corresponds to the concentration of scFv yielding 50% of the saturating signal.

## 3 Results

### 3.1 Selection of anti-tetanus toxin scFvs from a naïve phage display antibody library

Recombinant His-tagged H_c_ was expressed in *E. coli* and purified using IMAC (immobilised metal affinity chromatography. A commercial scFv phagemid library (Cambridge Antibody Technology; now AstraZeneca-Medimmune) (Vaughan et al., 1996), was screened against plastic-immobilised H_C_. Round 2 output clones (540) were screened in an ELISA binding assay. From this, 53 individual phage-antibodies were isolated and found to be selective for recombinant H_c_ by ELISA (Supplementary data, Table 1). Recombinant fragments of H_c_N and H_c_C were also used in phage screening experiments. Eleven of the 53 scFv clones were specific for H_c_C (H_c_C clones) whilst 26 were specific for the H_c_N sub-domain (H_c_N clones). The remaining 16 scFv clones exhibited no measurable levels of binding to either H_c_N or H_*N*_C and were presumed to recognise junctional epitopes formed between the sub-domains or epitopes on either sub-domain that are conformationally dependent on the other domain being present. These were subsequently referred to as ‘H_c_J’ clones.

The H_c_C and H_c_J binding scFv clones (27 clones in total) were fingerprinted using *Bst* NI RFLP analysis which revealed 13 different diversity groupings (Supplementary Fig. 1). The 26 H_c_N binding clones were also fingerprinted to reveal 18 diversity groupings (Supplementary Fig. 1).

### 3.2 Epitope binding by competition phage ELISA

Five unique H_c_J, H_c_C and H_c_N clones (15 clones total), each belonging to different *Bst* NI fingerprint groupings were selected for further characterisation which was initiated with full DNA sequencing of the VH and VL domains of each clone. They were also compared in terms of overall expression levels, soluble localisation to the periplasm and culture supernatant as well as their propensity for display on phage, as reported separately (Scott et al., 2008).

The five H_C_N binding clones (termed clones N1 through N5), five H_C_C binding (termed C1 through C5) and five H_C_J binding clones (J1 through J5) were assessed in a competition binding assay in order to characterise the diversity of scFvs isolated during phage display in terms of epitopes targeted. This was likely to reveal clones which could be used in CRAbs. H_c_ was coated onto a 96-well plate at 45 nM, the plate blocked and then pre-incubated with soluble non-phage-fused scFv at an approximate concentration of 3–5 μM. Purified phage-scFv fusion was then added to the plate without removing the soluble scFv. The detection step was *via* the phage coat protein using an anti-M13 HRPO immunoconjugate. Thus each pair-wise combination of phage-scFv and soluble scFv was assayed. Each phage-scFv clone was also assayed against itself in soluble form as an intrinsic positive control for competitive measurement. Parameters including optimal scFv and H_C_ concentrations as well as phage-antibody dilution were determined prior to conducting the full matrix of experiments at fixed concentrations (data not shown). Clone J3 exhibited extremely low levels of binding in phage fusion and non-fused format and this hindered its analysis in pair-wise competition binding experiments.

**Fig. 2:**
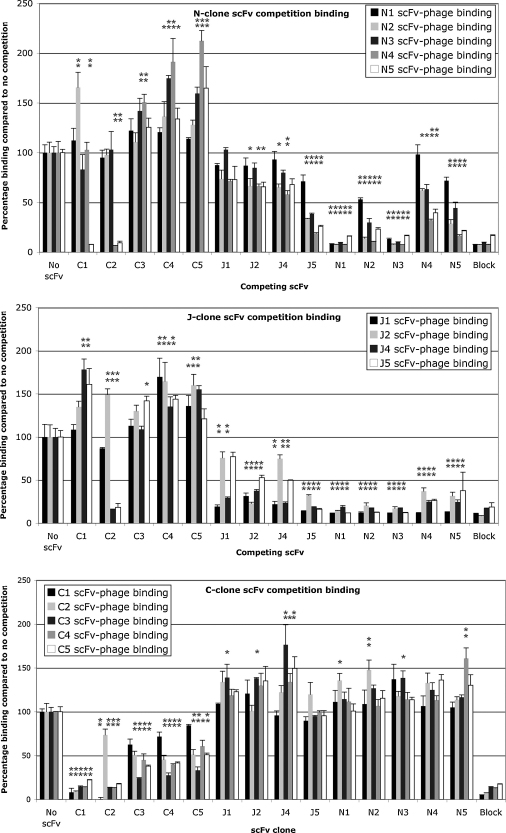
Anti-tetanus scFv epitope binning analysis. Each of the 14 phage-scFv fusions C1-C5, J1-J5 (excluding J3) and N1-N5 were assayed for binding to TT-H_c_ systematically at pre-determined fixed dilutions in the absence of soluble scFv and in the presence of each of the 14 scFvs in soluble non-phage-fused form (3–5 μM). Each phage-scFv was assayed against its non-fused form as an intrinsic positive control for competitive measurement. Each condition was measured in triplicate and bars are means with standard error bars. One-way ANOVA was used to determine whether there was significant global variation between phage-binding in the absence of scFvs and in the presence of scFvs for each clone. Significant variation was confirmed in each case (*p* < 0.01). A post-ANOVA pair-wise multi-comparative test was performed using the Dunnett algorithm which compares mean individual binding measurements with the positive binding control for each clone taking into account global background variation. Significant differences to the no scFv control are summarised in Table 1 and are denoted in this figure with significance of *p < 0.05 or **p < 0.01.

Fig. 2 and Table 1 show the results for all 14 scFvs that were assayed indicating the competition status in both orientations for each scFv. A one-way ANOVA was conducted followed by a Dunnett multiple comparison analysis used to identify statistically significant differences in phage-binding in the presence of each scFv compared to a control consisting of phage-scFv alone (100% binding). With few exceptions, the data revealed that all H_c_J and H_c_N clones compete for binding to TeNT-H_c_, both within and between groups. All H_c_C clones compete with each other for binding. With the exception of pairings C1 + N5 and C2 + (J4/J5/N4/N5), no soluble H_c_C clone was able to reduce binding of any H_c_J or H_c_N phage fusion and vice versa indicating that H_c_C clones were able to simultaneously bind to H_c_ with H_c_J or H_c_N clones except stated parings.

Interestingly, as shown in Fig. 2 some non-competitive scFv pairings demonstrated an apparent synergy in binding. A pronounced example being phage-scFv N4 which seems to exhibit a doubling in binding signal in the presence of scFvs C4 and C5.

### 3.3 Oligomerisation analysis

**Fig. 3:**
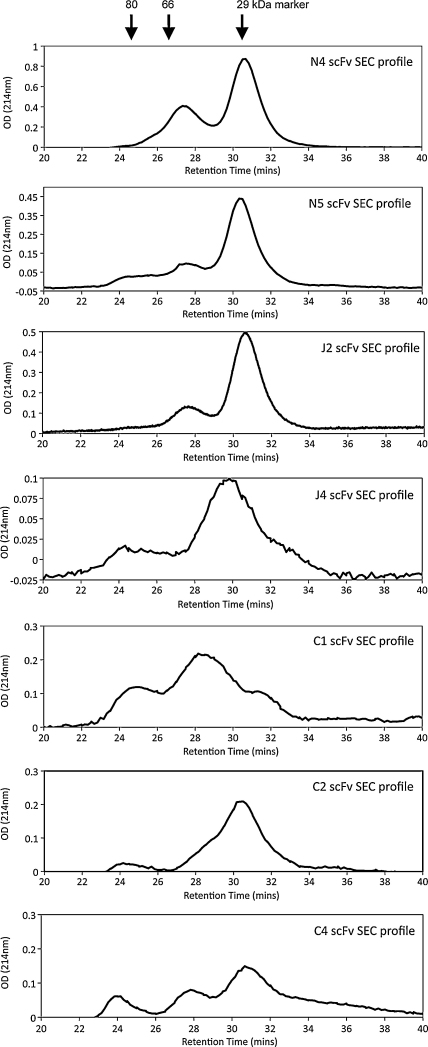
Chromatograms for the fractionation of seven anti-TT-H_c_ scFvs by size exclusion chromatography. Pooled IMAC eluate, containing semi-purified protein of anti-TT-H*c* scFvs C1, C2, C4, J2, J4, N4 and N5 was loaded onto a Superdex-75 gel filtration column and fractionated in 0.25 ml volumes using PBS pH7.4 as running buffer. Absorbance was continually measured at 214 nm. For purposes of clarity partial chromatograms are shown highlighting retention times from 20 to 40 min covering the possible apparent MW range for monomeric scFv and potential multimers.

The seven scFv clones C1, C2, C4, J2, J4, N4 and N5 were purified by cobalt IMAC followed by size exclusion chromatography in order to analyse the oligomerisation state of each scFv clone, isolate the monomeric form for affinity and potency determination and remove any residual contaminant proteins. A composite plot is shown in Fig. 3, which indicates varying levels of monomeric, dimeric and higher MW scFv polymers or aggregates. The gel filtration data is summarised in Table 2. Generally, with the exception of scFv C1 which was shown to be 80% dimeric, all clones were predominantly monomeric, however this did range from 90% monomer for scFv J4 down to 63% monomer for scFv N4.

### 3.4 *H*_c_ scFv affinity by ELISA

ELISA was used to determine *K*_D_ values for each scFv clone where possible, using biotinylated H_c_ in an avidin-capture assay. The *K*_D_ values determined (Table 3) varied considerably from clone to clone. The highest affinity clones were C1 and C4 with apparent *K*_D_ values of 26 nM and 36 nM respectively. The affinities of J4, N4 and N5 were around 10-fold less in comparison with *K*_D_ values of 301 nM, 196 nM and 171 nM respectively. It was not possible to determine a *K*_D_ value for clones C2 and J2 by ELISA method.

### 3.5 Hc scFv SPR kinetic measurements

Table 4 summarises the BIAcore data indicating best fit models and *Chi*^2^ values which indicate the closeness of the fit. Kinetic and affinity parameters are shown along with errors for the kinetic rates which are all at least 10-fold less than the parameter indicating excellent statistical reliability of the values. It was not possible to measure the affinity of clone C2 by surface plasmon resonance. There was no measurable change in RU when this clone was pulsed over the chip surface at the highest concentration 1200 nM for 3 min at a flow rate of 30 μl/min.

The SPR studies indicated that the highest affinity clones were C1, C4, J2 and N5 with apparent *K*_D_ values around the 10 nM value. In comparison the affinities of J4 and N4 were around 100 nM. The *K*_D_ values determined by BIAcore agreed fairly well with those determined by ELISA for scFv clones C1, C4, J4 and N4. The *K*_D_ values determined for clones J2 and N5 were at least 1000-fold and 10-fold lower respectively than values determined by ELISA. The BIAcore data for clones J2 and N5 was also used to calculate steady state affinity using scFv concentration vs *R*_eq_ plots (data not shown) which yielded *K*_D_ values of around 27.5 nM and 49.7 nM respectively concurring with the values obtained using kinetic parameters rather than ELISA results.

### 3.6 Potency (ganglioside binding inhibition) determination

The optimal concentration of monomeric TeNT-H_c_ for use in potency measurement assays was determined by titration. Immunosorbent plates were coated with the trisialoganglioside GT1b and blocked with 3% mPBS pH 7.4. The toxin was titrated from a concentration of 3 μM to 5 nM and incubated with the ganglioside coated wells. The *K*_D_ for binding of TeNT-H_c_ to the GT1b was 104 ± 16 nM. A TeNT-H_c_ concentration of 100 nM was chosen for use in toxin inhibition assays as this represents a limiting concentration of toxin with a sufficient assay window for accurately measuring binding inhibition. For scFv C1 it was possible to obtain a full dose–response curve and hence derive an IC_50_ value; however, this was not possible with the other clones due to their available concentrations being too low for their given potency. Therefore clones C2, C4, J2, J4, N4 and N5 were normalised to a concentration of 650 nM and measured at this concentration for comparative toxin inhibition.

**Fig. 4:**
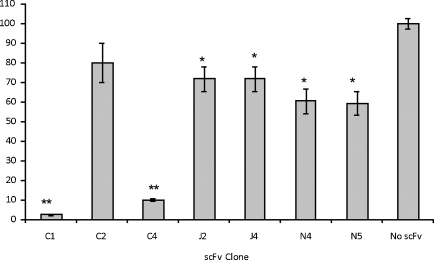
Comparative potency measurement for 7 anti-TT-H_c_ scFvs. The binding of recombinant monomeric TT-H_c_ to plate coated ganglioside GT1b was measured by ELISA at a fixed concentration (100 nM) both in the absence (“No scFv”) and the presence of the anti-TT-H_c_ scFvs C1, C2, C4, J2, J4, N4 and N5 at a single fixed concentration of 650 nM. Data is represented as % TT-H_c_-binding in comparison to binding in the absence of scFv which is normalised to 100%. Bars represent means of pentuplicate measurements with standard error. Binding in the presence of scFv was compared to binding in their absence using Student's *t*-test. *p < 0.05; **p < 0.01. No star = not significant.

Clone C1 inhibited binding of TeNT-H_c_ to the ganglioside in a dose-dependent manner with binding reduced to background levels at the highest concentration of scFv (900 nM). The dose-response curve generated resulted in an IC_50_ value of 212 ± 17 nM. Neutralising activity of the remaining clones was determined at a single fixed concentration of 650 nM allowing a comparative analysis (Fig. 4). Clone C1 completely ablated binding of TeNT-Hc to ganglioside (*p* < 0.01) whilst clone C4 diminished binding to around 10% of the levels observed in the absence of scFv (*p* < 0.01). Clones J2 and J4 reduced toxin–ganglioside binding to around 70% of levels in the absence of scFv (*p* < 0.05) and clones N4 and N5 reduced binding to around 60% (*p* < 0.05). Clone C2 did not reduce the binding of TeNT-H_c_ at the concentration tested.

## 4 Discussion

We have shown here that over 50 different scFvs against the heavy chain of the tetanus toxin can be selected from a human scFv phagemid library. It is unlikely that this library was naive with respect to tetanus toxin given that the individuals donating their blood used to construct this library would have been immunised against tetanus in childhood. This may explain the large number of diverse clones identified. From this, 15 were chosen and used in an investigation of phage display propensity (Scott et al., 2008). In the present study, the same 15 scFvs were used in a competition binding experiment in order to identify clones which could be used to make chelating recombinant antibodies (Neri et al., 1995; Wright and Deonarain, 2007; Scott, 2008, PhD thesis). Competition experiments revealed that the binding of one scFv could influence the binding of another, which would have major implications for bi-valent/bi-specific binding.

The competition binding assay that was utilised in this study relied on scFv-phage particles out-competing soluble scFvs bound to immobilised tetanus toxin (Fig. 2). Clearly non-overlapping epitope binders can confidently be identified this way from a positive binding signal, but negative binding could lead to false conclusions due to steric hindrance from the phage body or negative cooperativity mediated by the destruction of the second scFv epitope by conformational changes induced by the binding of the first scFv. However, many pair-wise clone comparisons showed non-overlapping binding in either orientation, thus making candidates suitable for a CRAb. Competition only seen for one orientation of a scFv pair could be due to significant differences between the affinity of the two clones. For example C1 (ELISA *K*_D_ = 26 nM) and N5 (ELISA *K*_D_ = 171 nM) exhibited competition for binding when binding of phage-scFv N5 was measured in the presence of pre-incubated soluble scFv C1 which resulted in a significant reduction in N5 binding. This was not observed in the alternative phage-scFv vs scFv orientation. As predicted, the majority of the H_c_C-binding scFvs were able to bind simultaneously with the H*c*N binders, with a small number of exceptions possibly due to steric clashes rather than recognition of the same or overlapping epitopes. The junctional epitope binders (H_c_J) were also able to bind simultaneously with the H_c_C binders but not H_c_N binders suggesting that there were also steric clashes or negative cooperativity effects upon either clone binding in the latter pairings.

Apparent positive cooperativity was an interesting observation in this study where the binding of one scFv enhanced the binding of a second scFv targeting a non-overlapping epitope (Fig. 2). This was seen for many pairings and in some cases both orientations (for example between C4 and J4). It is quite possible that binding of one clone results in a change in tertiary conformation of TeNT-H_c_ leading to a more favourable conformation of the cognate epitope of the second scFv clone and so making the interaction more thermodynamically and entropically favourable resulting in a higher affinity interaction. Such cooperative binding effects have been observed previously with three antibodies binding to non-overlapping epitopes of botulinum toxin (Marks, 2004; Nowakowski et al., 2002) and was also reported regarding antibodies against the whole tetanus toxin (Volk et al., 1984; Ziegler-Heitbrock et al., 1986). These observations also concur with Greenspans theory that cooperative binding effects are central to molecular recognition (Greenspan, 2001; Greenspan and Cooper, 1993) and are caused by conformational changes in the protein upon binding of one ligand which thermodynamically and entropically favour binding of the next. These observations have implications for the design of bi-valent or bi-specific scFv in that such binders may best be selected together as bi-specific pairs rather than individual scFvs which are later paired up. This also implies that the increase in affinity of such bi-valent pairs could be higher than the chelate model predicts for CRAbs as it assumes component scFvs bind independently (Zhou, 2003).

Clone J3 was found in initial studies to bind weakly to plate-immobilised H_c_ but not H_c_C or H_c_N fragments, hence leading to the “junctional” epitope binder designation. The weak binding to plate bound H_*C*_ did lead to poor amenability to the competition binding assays due to a low assay window for competing off J3 binding with other clones. It was subsequently found that the clone bound much more strongly to biotinylated H_*C*_ that was captured on plate bound avidin (data not shown) however the competition binding assay was optimised for H_c_ directly coated to an immunosorbent plate.

A sub-set of seven scFvs possessed properties which would facilitate construction, use and understanding of an anti-tetanus toxin CRAb library. These 7 scFvs underwent additional characterisation to include elucidation of affinity, neutralisation potency and oligomerisation behaviour which revealed many interesting features.

The degree of scFv multimerisation varied between clones despite having an identical inter-domain linker, which normally influences scFv oligomerisation status (Perisic et al., 1994). The fact that an identical light chain is present in scFv clones C1 and C2 implies that the differences in dimerisation (80% for C1 vs 25% for C2) and monomeric affinity (<50 nM for C1 vs >1000 nM for C2) are heavy chain driven. This latter observation is consistent with previous observations that the VH domain contributes significantly in antibody antigen interactions (Ohno et al., 1985); in particular the CDR3 region (Collis et al., 2003).

The range of affinities as determined by immuno-assay and surface plasmon resonance were broad (10 nM to over 1000 nM). However, many high affinity binders were successfully isolated (e.g. clones C1 and C4; Tables 3 and 4). Generally, the affinities determined by the two independent methods correlated well (e.g. scFv C1) but with major differences observed for clones J2 and N5. It is not unusual to observe differences in affinities between different measurement techniques (Kaufman and Jain, 1992). One may hypothesise that these observations may be due to differential presentation of the antigen in both assay formats. In the ELISA format, TT-H_c_ is anchored *via* surface biotins to avidin molecules which are directly coated onto the plate surface. Thus the toxin is still relatively proximal to the plate surface. However, on a BIAcore sensor surface, Hc is bound to streptavidin which is itself covalently coupled to a carboxymethylated dextran matrix which is at least 100 nm from the solid surface. In the BIAcore situation, access to antigenic epitopes is more favourable than in the ELISA method since it is more comparable to true solution interaction. The BIAcore system is also a “flowing” system, not static as in ELISA and so perhaps more similar to a physiological situation. Kinetic constants will certainly be different.

The BIAcore data for most clones fit to the 1:1 Langmuir binding model as theoretically expected. However, clones J4 and N4 produced the best model fit when using the two-state conformational change model which has been used in previous SPR reports (Carrick et al., 2001; Townsend et al., 2006). These clones exhibited distinct bi-phasic dissociation kinetics. The conformational change model accounts for an analyte binding to a ligand producing a ligand–analyte complex which undergoes a conformational change that reverts upon dissociation of the analyte. It is plausible that this observation is in line with the other observations discussed below which centre around cooperative binding effects and toxin neutralisation effects mediated by conformational changes in the toxin upon binding of a scFv. It may also be hypothesised that the bi-phasic nature of the dissociation of clones J4 and N4 are attributable to the presence of a monomer–dimer mix caused by re-equilibration of the SEC isolated monomer. It was not possible to fit the heterogeneous analyte model due to the fact the absolute amounts of monomer and dimer were unknown.

The affinity values determined here generally compare favourably to the affinities determined by Poulsen et al. (2007) who looked at the immunological response to tetanus toxoid in humans (Poulsen et al., 2007). The *k*_off_ rates seen for the 7 scFvs here fall within the median range identified by Poulsen. Observed differences may be due to the loss of the original VH-VL pairings in the library used in the present study. A full study of the relationship of scFv affinity and ganglioside neutralisation potency was not possible due to some of the poor affinities observed with certain clones. Using a fixed concentration of scFv, we could show that generally, the higher the affinity, the more effective the blocking (Fig. 4). This is in agreement with work on other toxins such as botulinum toxin (Levy et al., 2007) and anthrax toxin (Maynard et al., 2002).

Interestingly, in this study we found that four out of five H*c*N binding clones reduced toxin binding to the ganglioside, although they do not recognise the H_c_C sub-domain that is known to bind ganglioside (Fotinou et al., 2001). In fact 14/15 of the clones described here inhibited toxin neutralization (data not shown). These observations may link in with those made in the competition binding studies whereby certain scFv combinations exhibited synergy in binding likely to be caused by conformational changes in the toxin upon binding of one scFv that favour binding of the next (Greenspan, 2001). From this we may postulate that scFvs binding at a site on the toxin distal to the ganglioside binding site may indirectly alter the structure of that site and thus hinder its interaction with the ganglioside. Thus generally speaking, the epitope targeted by an antibody may not be an important factor when identifying toxin neutralising antibodies. It is evident that affinity may be the more important factor when comparing two scFvs binding the same region. For example C1 and C2 bind to overlapping epitopes, but C1 is the more potent inhibitor due to its 1000-fold higher affinity.

Based on the diversity, display propensity (Scott et al., 2008), epitopes bound and affinities, the seven clones (C1, C2, C4, J2, J4, N4 and N5) were deemed appropriate for further study as components in bi-specific scFvs known as CRAbs (Scott, 2008, PhD thesis).

## Figures and Tables

**Supplementary Fig. I:**
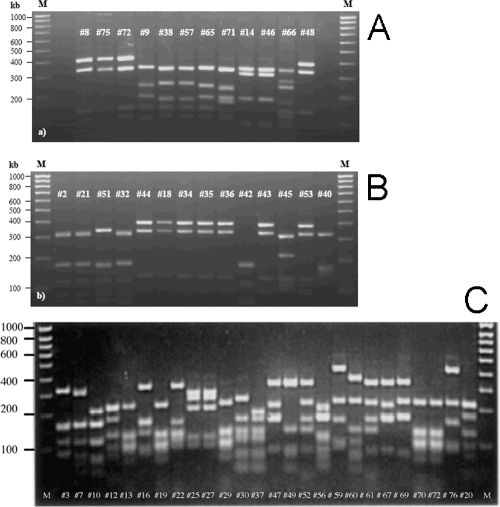
*Bst* NI fingerprint analyses of scFv clones. *Bst* NI RFLP analysis of scFv clones isolated from a phagemid library panned against TeNT-H*c* and verified as target specific by ELISA. The scFv genes were amplified from minipreped phagemid vector by PCR using primers LMB3 and FDSEQ1. The product was digested with *Bst* NI and then underwent electrophoretic separation on a 4% agarose gel stained with ethidium bromide. Marker is Hyperladder IV (Bioline). Gel A (HcC clones), Gel B (H*c*J clones) and Gel C (H*c*N clones). Clone numbers refer to original screening designation.

**Table 1:**
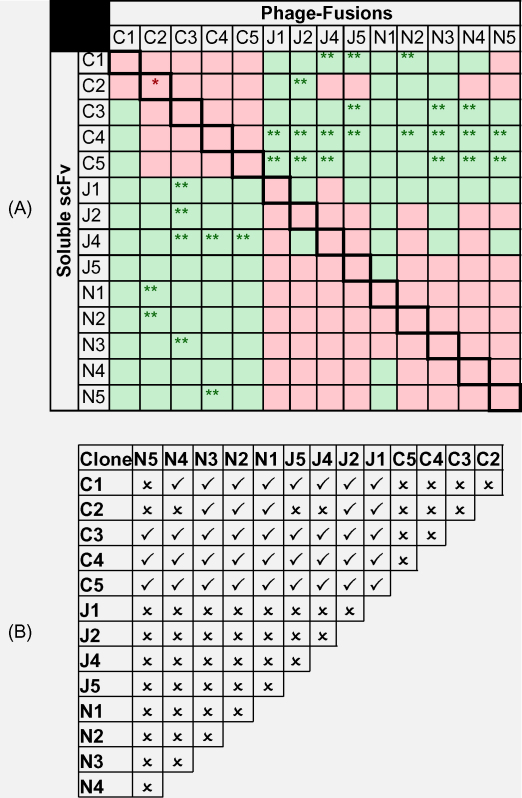
Summary of scFv competition binding analysis and chelating pair identification.

The competition binding analysis data generated for each phage-scFv fusion in turn is summarised in (A). Green squares indicate that a particular soluble scFv did not result in any statistically significant reduction in binding of the pair-wise assayed phage fusion. Red squares indicate that a particular soluble scFv resulted in a measurable and statistically significant reduction in binding of the pair-wise assayed phage-scFv fusion. *A reduction in binding of phage-scFv C2 in the presence of its own soluble form (as would be expected) but the *p*-value for the difference was 0.1 > *p* > 0.05 and so was modest. **Presence of a particular soluble scFv resulted in an increase in binding of the pair-wise assayed phage-scFv fusion above that of the phage-scFv alone (positive control) indicating a cooperative binding effect. The summary data from (A) was used to generate (B) which indicates whether a particular scFv pairing is chelating (non-competitive), illustrated with a tick (), or non-chelating (competitive), illustrated with a cross ().

**Table 2:** Summary of size exclusion chromatographic analysis of 7 anti-TT-H_c_ scFv clones.

Clone	Monomer			Dimer			Aggregates		
	RT (min)	MW (kDa)	%	RT (min)	MW (kDa)	%	RT (min)	MW (kDa)	%
C1	31.3	29.7	20	28.5	43.6	80	NA	NA	NA
C2	30.5	33.1	70	28.4	44	24	24.1	79.4	6
C4	30.66	32.4	65	27.83	48	20	24.01	80.5	15
J2	30.67	32.4	72	27.67	48.8	28	NA	NA	NA
J4	29.83	36	90	NA	NA	NA	24.3	77.3	10
N4	30.67	32.4	63	27.33	51	37	NA	NA	NA
N5	30.33	33.9	78	27.5	49.9	22	NA	NA	NA

The retention times (RT), apparent molecular weights (MW) and % dominance of the monomeric, dimeric and aggregate forms of each scFv clones are shown. NA = not applicable.

**Table 3:** Monomeric scFv stock concentrations and ELISA *K*_D_ values.

Clone	Stock concentration (nM)	Assay concentration range (nM)	*K*_D_ (nM)	Standard error of *K*_D_ (±nM)
C1	1800	1–500	26	1.4
C2	1200	1–600	>1000	NA
C4	1200	1–600	36	3.2
J2	3300	3–1650	>1000	NA
J4	6600	6.5–3330	301	7.5
N4	6600	6.5–3330	196	7.1
N5	3280	1.64–840	171	4

**Table 4:** Summary of surface plasmon resonance kinetics analyses.

Clone	Model	*Chi*^2^	$k_{\text{on}}^{1}$ (M^−1^ s^−1^)	SE-$k_{\text{on}}^{1}$ (M^−1^ s^−1^)	$k_{\text{off}}^{1}$ (s^−1^)	SE-$k_{\text{off}}^{1}$ (s^−1^)	$k_{\text{on}}^{2}$ (s^−1^)	SE-$k_{\text{on}}^{2}$ (s^−1^)	$k_{\text{off}}^{2}$ (s^−1^)	SE-$k_{\text{off}}^{2}$ (s^−1^)	*K*_D_ (M)
C1	A	0.463	1.61 × 10^4^	219	1.42 × 10^−4^	3.73 × 10^−7^	n/a	n/a	n/a	n/a	8.82 × 10^−9^
C4	A	0.938	2.32 × 10^4^	103	3.11 × 10^−4^	7.28 × 10^−7^	n/a	n/a	n/a	n/a	1.34 × 10^−8^
J2^a^	A	0.45	1.41 × 10^4^	323	3.68 × 10^−4^	1.07 × 10^−5^	n/a	n/a	n/a	n/a	2.61 × 10^−8^
J2^b^	B	24.5	7.9 × 10^4^	626	2.58 × 10^−3^	1.04 × 10^−4^	3.98 × 10^−3^	1.91 × 10^−4^	1.83 × 10^−3^	4.3 × 10^−5^	1.00 × 10^−8^
J2^b^	A	23.3	7.67 × 10^4^	682	7.10 × 10^−4^	2.41 × 10^−6^	n/a	n/a	n/a	n/a	9.25 × 10^−9^
J4	B	9.42	5.22 × 10^4^	403	1.65 × 10^−2^	1.28 × 10^−4^	1.94 × 10^−3^	2.11 × 10^−5^	2.48 × 10^−3^	2.28 × 10^−5^	1.77 × 10^−7^
N4	B	0.25	1.61 × 10^4^	312	1.81 × 10^−2^	1.67 × 10^−4^	2.24 × 10^−3^	1.53 × 10^−5^	3.8 × 10^−4^	1.43 × 10^−5^	1.63 × 10^−7^
N5^a^	A	0.09	1.34 × 10^4^	325	7.43 × 10^−4^	5.2 × 10^−6^	n/a	n/a	n/a	n/a	5.55 × 10^−8^
N5^b^	A	6.59	1.98 × 10^4^	6.25	2.66 × 10^−4^	4.99 × 10^−7^	n/a	n/a	n/a	n/a	1.34 × 10^−8^

Model A: Langmuir 1:1 binding. Model B: two-state reaction with conformational change.

a Low *R*_max_ surface.

b Moderate *R*_max_ surface.

## Appendix A Supplementary data

Supplementary Table 1 Anti-tetanus phage display selection input and output titres.
