# Supplementary material for: Characterisation of a panel of anti-tetanus toxin single-chain Fvs reveals cooperative binding
Source: Mol Immunol. 2010 Jun;47(10):1931–41. doi: 10.1016/j.molimm.2010.02.020 (PMC2874839; doi:10.1016/j.molimm.2010.02.020)
Supplement: Supplementary Table 1 — Anti-tetanus phage display selection input and output titres. [file mmc1.pdf]

Supplementary Table 1.

Input titre (pfu/ml)	Elution method	Output titre (pfu/ml)	% binders
3.5×10^{10}	Glycine	1.2×10^{6}	9.1
	TEA	1.2×10^{5}	12.5
	TG1 cells	1.2×10^{3}	14.8
5.0×10^{11}	Glycine	1.7×10^{6}	38.5
	TEA	2.2×10^{5}	13.6
	TG1 cells	2.0×10^{3}	1.1
